# Long-term prenatal exposure to paracetamol is associated with DNA methylation differences in children diagnosed with ADHD

**DOI:** 10.1186/s13148-017-0376-9

**Published:** 2017-08-02

**Authors:** Kristina Gervin, Hedvig Nordeng, Eivind Ystrom, Ted Reichborn-Kjennerud, Robert Lyle

**Affiliations:** 1 Department of Medical Genetics, Oslo University Hospital and University of Oslo, Oslo, Norway; 2 PharmaTox Strategic Research Initiative, Faculty of Mathematics and Natural Sciences, University of Oslo, Oslo, Norway; 3 Pharmacoepidemiology and Drug Safety Research Group, Department of Pharmacy, School of Pharmacy, University of Oslo, Oslo, Norway; 4 Department of Child Health, Norwegian Institute of Public Health, Oslo, Norway; 5 Department of Mental Disorders, Norwegian Institute of Public Health, Oslo, Norway; 6 Department of Psychology, University of Oslo, Oslo, Norway; 7 Institute of Clinical Medicine, University of Oslo, Oslo, Norway

**Keywords:** EWAS, DNA methylation, Paracetamol, ADHD, Epidemiology, MoBa

## Abstract

**Background:**

Epidemiological studies have shown that long-term exposure to paracetamol during pregnancy is associated with attention-deficit/hyperactivity disorder (ADHD). The mechanism by which paracetamol may modulate the increased risk of developing ADHD is currently unknown. We have conducted an epigenome-wide association study (*n* = 384 cord blood samples) and investigated whether prenatal exposure to paracetamol is associated with DNA methylation in children diagnosed with ADHD.

**Results:**

Analyses identified significant differences in DNA methylation (*n* = 6211 CpGs) associated with prenatal exposure to paracetamol for more than 20 days in children diagnosed with ADHD compared to controls. In addition, these samples were differentially methylated compared to samples with ADHD exposed to paracetamol for less than 20 days (*n* = 2089 CpGs) and not exposed to paracetamol (*n* = 193 CpGs). Interestingly, several of the top genes ranked according to significance and effect size have been linked to ADHD, neural development, and neurotransmission. Gene ontology analysis revealed enrichment of pathways involved in oxidative stress, neurological processes, and the olfactory sensory system, which have previously been implicated in the etiology of ADHD.

**Conclusions:**

These initial findings suggest that in individuals susceptible to ADHD, prenatal long-term exposure to paracetamol is associated with DNA methylation differences compared to controls.

**Electronic supplementary material:**

The online version of this article (doi:10.1186/s13148-017-0376-9) contains supplementary material, which is available to authorized users.

## Background

Attention-deficit/hyperactivity disorder (ADHD) is a common childhood disorder defined by inattention, hyperactivity, and impulsivity, which impair normal functioning and development. It is one of the most prevalent psychiatric disorders of childhood affecting approximately 5% of children worldwide, often continuing into adulthood [1]. ADHD is considered to have a strong genetic basis, with heritability estimates up to 70% [2]. Although candidate gene studies have identified genes associated with ADHD [2], numerous genome-wide association studies (GWAS) [3–9] and meta-analyses [10] have not found any significant ADHD risk variants [11].

Several epidemiological studies have shown that exposure to a range of putative environmental risk factors are associated with ADHD, particularly during the prenatal and perinatal period. However, these factors can only be regarded as correlates because, at present, convincing evidence that the associations reflect causal relationships have not been established [12]. ADHD is a complex disorder influenced by the interplay of various genetic and environmental factors. The mechanisms by which the environment mediates ADHD susceptibility is not well understood, but epigenetic changes may be involved [13–17].

Recently, five epidemiological studies have revealed a consistent association between long-term exposure to paracetamol (acetaminophen) during pregnancy and ADHD symptoms [18–21] and adverse neurodevelopment in children [22]. Paracetamol, which is available without prescription, is the most commonly used drug during pregnancy taken by ~50% of pregnant woman to treat fever, headache, and other pain conditions [23]. If the association between prenatal paracetamol exposure and neurodevelopmental disorders is causal, the underlying mechanisms remain to be clarified.

In this study, we aimed to assess whether epigenetic differences are associated with prenatal exposure to paracetamol and development of ADHD. To do this, we have conducted an epigenome-wide association study (EWAS) in samples selected from the Norwegian Mother and Child Cohort (MoBa) and investigated whether long-term prenatal exposure to paracetamol was associated with DNA methylation changes in cord blood and clinical ADHD diagnoses recorded in the Norwegian Patient Registry (NPR) from 2008 to 2014.

## Results

### Neither paracetamol nor ADHD alone is associated with DNA methylation differences

The samples in this study were selected to enable a systematic investigation of the association between DNA methylation and paracetamol exposure in children with and without ADHD (Table 1). First, we stratified the analyses on paracetamol exposure and ADHD separately to evaluate the associations with DNA methylation. Pairwise analyses of the groups exposed to paracetamol (drug exposure group) or diagnosed with ADHD (ADHD control group) alone compared to control samples revealed no significant differences in DNA methylation (FDR <0.05, data not shown).

**Table 1:** Sampling and study design

Group	Type	No.	Covariates				Outcome
			Sex M/F	Smoking: never/daily/sometimes (days)	Maternal age (median age)	Gestational age (median weeks)	
No Paracetamol, no ADHD	Control group	96	52/44	72/0/4	29.5	40	DNA methylation
Paracetamol, no ADHD	Drug exposure group						
Sporadic^a^		78	33/45	65/2/2	31.0	40	
Long term^b^		18	10/8	14/0/2	28.0	39	
No Paracetamol, ADHD	ADHD control group	96	66/30	73/0/11	29.0	40	
Paracetamol, ADHD	Synergy group						
Sporadic^a^		77	57/20	58/0/10	29.0	40	
Long term^b^		19	14/5	13/0/3	29.0	39	

^a^6–19 days

^b^≥20 days

### Long-term paracetamol exposure is associated with DNA methylation differences in children with ADHD

Next, we investigate the synergistic effect of prenatal exposure to paracetamol on DNA methylation differences in children with ADHD. To do this, we stratified the analyses on samples exposed to paracetamol (synergistic effect group, Table 1) and controls. Further, we performed comparisons of the synergistic group to the ADHD control group and the drug exposure group to enable interpretation of the associations.

**Fig. 1:**
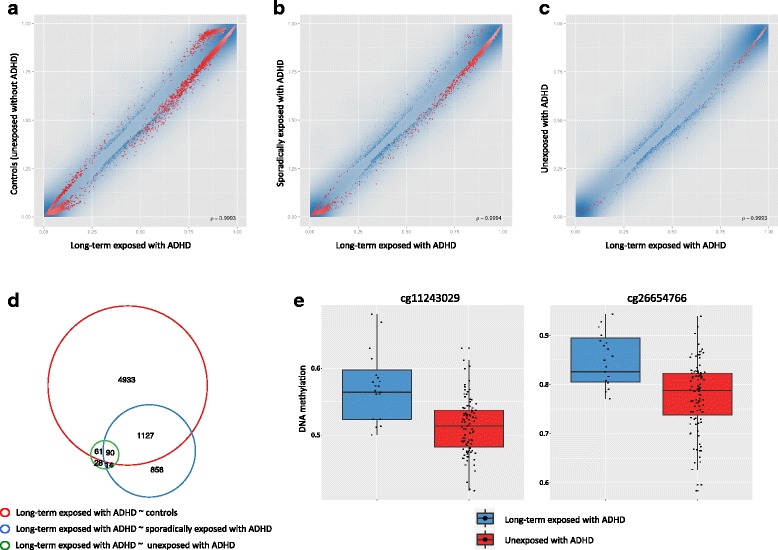
Group-wise differences in DNA methylation. Scatter plots of DNA methylation differences between **a** long-term exposed samples with ADHD (*n* = 19) and controls (*n* = 96), **b** long-term exposed samples with ADHD (*n* = 19) and sporadically exposed samples with ADHD (*n* = 77), and **c** long-term exposed samples with ADHD (*n* = 19) and unexposed samples with ADHD (*n* = 96). Each *point* represents a CpG site (*n* = 390,787), and significant CpGs are marked in *red* (BH FDR <0.05). **d** Venn diagram summarizing the overlap of CpGs that were differentially methylated between the three groups. **e** Example *box plots* showing the DNA methylation levels at two representative CpGs that were differentially methylated between long-term exposed samples with ADHD and unexposed samples with ADHD

Comparison of the samples exposed to paracetamol with ADHD (synergistic effect group) to each of the other three groups did not identify any differential DNA methylation below the significance threshold (FDR <0.05, data not shown). However, analyses contrasting sporadic and long-term paracetamol use revealed that exposure to paracetamol for ≥20 days was associated with DNA methylation differences in samples with ADHD. These samples showed differential DNA methylation compared to the control group after FDR correction in a large number of CpG sites (*n* = 6211 CpGs, mean = 0.012, range = −0.28 to 0.20; Fig. 1a, Additional file 1: Table S1). The 6211 CpGs were 90% Winsorized in the long-term exposed samples with ADHD to investigate the potential influence of outliers. The results from this analysis showed that a small proportion of the CpGs (*n* = 697, 11.2%) were driven by outliers.

The long-term exposed samples with ADHD were also differentially methylated compared to exposed samples with ADHD in both the sporadic (*n* = 2089 CpGs, mean = 0.025, range = −0.13 to 0.17; Fig. 1b, Additional file 2: Table S2) and unexposed samples with ADHD (*n* = 193 CpGs, mean = 0.029, range = −0.11 to 0.17; Fig. 1c, Additional file 3: Table S3). There was substantial overlap between the differentially methylated CpGs identified by these three comparisons (Fig. 1d). Quantile-quantile (Q-Q) plots of the observed versus expected *p* values and corresponding histograms of *p* values for the three comparisons above show strong inflation (Additional file 4: Figure S1). Here, the observed quantiles are consistently higher than their expected values under the null hypothesis of no association between long-term exposure to paracetamol and DNA methylation differences in children with ADHD to each of the three comparisons (lambda ranging from 1.7 to 2.2). We performed surrogate variable analysis (SVA) to detect possible hidden covariates with a global effect on DNA methylation between the groups. No significantly associated surrogate variables were detected after correction for the covariates already included in the statistical model (i.e., cell-type composition using a suitable reference panel based on cord blood, gestational age, maternal age, smoking, and bisulfite conversion). Hence, these results suggest that there are no unmeasured covariates with a strong confounding effect on the significantly associated DNA methylation differences.

We could not detect any effect due to use in different trimesters, but common for the samples in the high paracetamol exposure group was that they were exposed to paracetamol in at least two of the three trimesters, usually across all trimesters.

The majority of the significant CpGs between groups involved very small differences in DNA methylation. To increase the likelihood of biological relevance of the differences in DNA methylation being related to ADHD and paracetamol exposure, we chose to focus the downstream analyses on the CpGs displaying both statistical significance and a large effect size (combined score, see the “Methods” section). Interestingly, top ranked CpGs overlapped genes previously linked to ADHD (*SGTB* [24] and *CADM1* [25]). In addition, genes involved in neural development (*REST* [26], *ZNRF1* [27], *RabGEF1* [28], and *CUX1* [29]), neurotransmission (*SYN2* [30] and *DDAH1* [31]; Fig. 1e (left)), noradrenergic neurotransmitter system (*PHOX2A*), voltage channels (*KCNJ8* and *CACNG8* [32]), and peroximal lipid metabolism (*PHYH*; Fig. 1e (right)) clustered among the top ranked genes, all of which have been implicated in neurodevelopmental disorders. Regional analyses did not identify any significant differential DNA methylation between groups.

### Enrichment of pathways involved in oxidative stress and the olfactory system

Gene ontology (GO) analyses of the top 100 significantly differentially methylated genes ranked on the combined score identified enrichment of pathways involved in oxidative stress among the genes showing increased DNA methylation (Additional files 1, 2, and 3: Tables S1–3). This enrichment was more prominent among the differentially methylated genes between samples with ADHD, but discordant for paracetamol exposure (Table 2). In addition, receptor-mediated endocytosis, vesicle-mediated transport, and bicarbonate transport pathways, which are involved in the neurotransmission, were enriched. Genes showing decreased DNA methylation were enriched for pathways related to the olfactory system and neurological system processes. The olfactory system and perception of smell were highly and consistently enriched among the top 100 genes identified in all the three comparisons (Table 2).

**Table 2:** Gene ontology results based on top 100 ranked according to significance and effect size

Category	Term	Count	Size	*P* value
Increased DNA methylation				
GO:0042744	Hydrogen peroxide catabolic process	7	22	1.0 × 10^−8^
GO:0072593	Reactive oxygen species metabolic process	4	209	2.1 × 10^−4^
GO:0015671	Oxygen transport	2	15	2.1 × 10^−5^
GO:0006898	Receptor-mediated endocytosis	4	235	3.2 × 10^−4^
GO:0016192	Vesicle-mediated transport	7	877	3.3 × 10^−4^
GO:0042542	Response to hydrogen peroxide	3	94	3.5 × 10^−4^
GO:0016192	Bicarbonate transport	2	32	8.1 × 10^−4^
GO:0006979	Response to oxidative stress	8	198	7.4 × 10^−3^
GO:0055114	Oxidation-reduction process	5	837	7.2 × 10^−3^
Decreased DNA methylation				
GO:0050911	Detection of chemical stimulus involved in sensory perception of smell	8	248	1.0 × 10^−8^
GO:0009593	Detection of chemical stimulus	6	317	1.0 × 10^−8^
GO:0050906	Detection of stimulus involved in sensory perception	8	325	1.0 × 10^−8^
GO:0007606	Sensory perception of chemical stimulus	6	344	1.0 × 10^−8^
GO:0050877	Neurological system process	6	899	2.9 × 10^−3^
GO:0004984	Olfactory receptor activity	6	247	1.0 × 10^−8^

## Discussion

We have studied DNA methylation differences associated with prenatal exposure to paracetamol and ADHD in cord blood samples selected from a large prospective birth cohort. This is the first study to investigate the recent pharmacoepidemiological findings of increased risk of ADHD after long-term prenatal exposure to paracetamol. In the pairwise comparisons of the groups, only the group with long-term exposure to paracetamol and ADHD showed differential DNA methylation. These results indicate a dosage effect of paracetamol on the observed differences in DNA methylation. This is in agreement with the epidemiological link identified in five large recent studies [18–22]. The same effect has been observed in adult mice where long-term exposure to paracetamol, but not ibuprofen [33], during neonatal brain development was associated with cognitive functions [34].

Several studies, based on selected candidate genes [13, 14, 16] and two recent EWAS [15, 17], have revealed an association between DNA methylation differences and ADHD symptoms. No differential DNA methylation associated with ADHD alone was identified in our study. Several factors involving variations in the sample size, criteria underlying the ADHD phenotype, study design, type of tissue, time point, and confounding factors are likely to explain the lack of replication in our study (or between any of the other studies). Most of the studies are based on either peripheral whole-blood or saliva samples from older children. Hence, the associated differences in DNA methylation are likely to also involve different environmental influences during childhood and having tissue-specific roles. The recent EWAS by Walton et al. [17] is perhaps the most comparable to our study in terms of the time point and tissue investigated. Cord blood, which is sampled at the time of birth, reflects prenatal environmental influences on the development of ADHD. However, we did not replicate any of the 13 CpGs reported in this study.

A large number of differentially methylated CpGs were identified in this study, particularly between samples with ADHD and long-term exposure to paracetamol and controls (unexposed without ADHD). The majority involved small differences in DNA methylation. Sensitivity analyses showed that outliers in this group influenced a small proportion (11.2%) of the differentially methylated CpGs. It is not possible to differentiate the influence of technical outliers from extreme DNA methylation values reflecting paracetamol dosage-effect (high number of days of exposure) associations. To increase biological relevance, the analyses and interpretation of the results were based on the genes displaying significant and the largest differences in DNA methylation. Several of the top ranked genes have previously been linked to not only ADHD but also neural development and neurotransmission. These are categories involved in the brain development and function and are also enriched among genes genetically associated with ADHD [35]. ADHD is a complex disorder, which involves many genes interacting within multiple pathways, also epigenetically dysregulated genes. Pathways are interconnected, and a defect in one may lead to dysfunction in others, potentially leading to a high number of epigenetically dysregulated genes. The top 100 genes with the largest DNA methylation differences in our study were enriched in pathways involved in oxidative stress, neurological system processes, and the olfactory system. Several studies have detected altered levels of oxidative stress in different tissues in ADHD patients, including the blood [36], saliva [37], and serum [38]. This has also been observed in autism spectrum disorders [39]. This could suggest that oxidative stress is a peripheral biomarker of ADHD or that ADHD is a systemic abnormality with consequences in the brain. The brain is particularly susceptible to oxidative stress due to a high level of oxygen utilization and a modest antioxidant defense [40]. Interestingly, oxidative damage and redox imbalance have also been suggested as one of the paracetamol toxicity mechanisms [41]. Further, pathways related to the olfactory system were highly enriched among the genes displaying decreased DNA methylation. The olfactory system is responsible for the detection of smell. Activated olfactory receptors induce a nerve impulse, which is ultimately transmitted to the brain. Deficits in the function of this system are a common feature in several neurodevelopmental disorders, including ADHD [42]. Specifically, increased odor sensitivity has been found in both children [43] and adults [44] with ADHD.

Interestingly, we identified more differentially methylated positions when comparing the long-term exposed samples to sporadic exposed samples than to unexposed samples with ADHD. Whereas this observation is difficult to explain, it could reflect the fact that dose-response relationships are usually not linear. Unfortunately, it is difficult to model and monitor a dose effect and timing during pregnancy due to the way the MoBa questionnaires were designed and completed. Future studies including a larger sample should investigate whether this trend remains.

The results presented in this study should be interpreted in light of several limitations. Although this study is based on one of the largest prospective birth cohorts in the world, the number of samples possessing both clinical ADHD diagnoses and long-term prenatal exposure to paracetamol was relatively small. Hence, the association between paracetamol and ADHD should be replicated in a larger set of samples. We report DNA methylation differences in cord blood samples, and whether these differences also reflect changes in the brain needs to be established. Although the analyses and results were focused on differentially methylated genes with large effect sizes, the effect on gene expression needs to be explored. Heritable impulsive traits are associated with paracetamol use during pregnancy [45]. Future studies should investigate the link found here while controlling for intergenerational transmission of impulsive traits.

There are also several strengths that should be taken into account. We used a clinical diagnosis of ADHD given by specialists obtained through linkage to the NPR. Although registry-based diagnoses of ADHD has been used as outcome in a number of previous Scandinavian studies on ADHD, including the abovementioned Danish birth cohort study of paracetamol use during pregnancy and ADHD [18], they have not been validated. The study is based on cord blood samples collected at birth, thereby eliminating the confounding of ADHD stimulant drugs, which are known to influence DNA methylation [46]. Moreover, as samples with maternal co-medication were excluded, there was no influence of other psychotropics or analgesics on DNA methylation. In addition, other known confounders were included as covariates. Importantly, correction for cord blood cell-type composition between groups was done based on a suitable cord blood reference data set, which we recently established [47].

## Conclusion

This study identified altered DNA methylation differences at genes involved in oxidative stress, neural transmission, and olfactory sensory pathways associated with long-term exposure to paracetamol during pregnancy in children diagnosed with ADHD. These results suggest that in individuals susceptible to ADHD, prenatal long-term exposure to paracetamol is associated with DNA methylation changes. That is, individuals susceptible to ADHD respond differently compared to controls to long-term paracetamol exposure during development. This is compatible with the epidemiological evidence of the increased risk of developing ADHD with long-term paracetamol exposure. Further, these results lend novel insights into the etiology of ADHD and may serve as disease biomarkers in blood. Future developments should also investigate the functional impact of these DNA methylation differences on neural development in model systems.

## Methods

### Study population

The samples were obtained from MoBa, which is a large prospective population-based pregnancy cohort study conducted by the Norwegian Institute of Public Health [48]. Participants were recruited from all over Norway between 1999 and 2008. The women consented to participation in 40.6% of the pregnancies. The cohort now includes 114,500 children, 95,200 mothers, and 75,200 fathers. Blood samples were obtained from both parents during pregnancy and from mothers and children (umbilical cord) at birth from approximately 90,000 participants [49, 50]. This study is based on Data Version 8 released by MoBa in 2015. The MoBa cohort was linked to the Medical Birth Registry of Norway (MBRN) and the Norwegian Patient Registry (NPR) using the women’s personal 11-digit identification number.

### Measures

#### Prenatal use of paracetamol

Information about paracetamol use (ATC code: N02BE01) was based on three questionnaires (Q1: from conception to gestational week 18, Q3: to gestational week 30, and Q4: to delivery). In order to minimize potential confounding by gestational age, only term children were included in the study (≥37 weeks). Samples with no reported co-medication together with paracetamol were selected to be certain that the number of days of use corresponded to paracetamol use. We defined long-term use as use of paracetamol for 20 days or more during pregnancy based on previous studies [18–22]. Moreover, trimester of paracetamol use was recorded and assessed in sub-analyses.

### Measure of ADHD

Information about the children’s ADHD diagnoses from 2008 to 2014 was obtained from the NPR. From 2008, all hospitals owned and funded by the government and outpatient clinics report individual-level diagnoses given by specialists according to the 10th revision of the International Classification of Disease (ICD-10). Children registered with an ICD-10 diagnosis of hyperkinetic disorder (HKD) (F90.0, F90.1, F90.8, or F90.9) between 2008 and 2013 were identified and defined as having ADHD. HKD requires the combination of inattentive and hyperactive symptoms and thus corresponds to the combined subtype in the Diagnostic and Statistical Manual (DSM) system [51, 52].

### Sampling and study design

The participants included in this study (*n* = 384) were selected to allow a cohort study design with 96 samples in each of the four categories (Table 1): (1) unexposed to paracetamol without ADHD (randomly selected control group), (2) exposed to paracetamol without ADHD (drug exposure group), (3) unexposed to paracetamol with ADHD (ADHD control group), and (4) exposed to paracetamol with ADHD (synergistic effect group). Selection criteria for paracetamol exposure and the clinical ADHD diagnose are described above. Potential covariates from MoBa and MBRN included infant sex, gestational age at delivery, maternal age, smoking, and alcohol consumption during pregnancy.

### DNA methylation analysis

#### Microarray preprocessing and quality control

Bisulfite conversion of 500-ng cord blood DNA was done using the EZ-96 DNA Methylation-Gold Kit (Zymo Research) according to the manufacturer’s instructions. The samples were randomly located on 96-well plates to minimize potential batch effects related to bisulfite conversion. DNA methylation status was assessed using the Infinium HumanMethylation 450 BeadChip (Illumina). All analyses were carried out using the R programming language (http://www.r-project.org/), and the raw data were preprocessed using the approach implemented in RnBeads v.1.2.1 [53] in two steps (before and after normalization). First, cross-reactive probes [54] (*n* = 29,233), probes with overlapping SNPs in any of the bases in the target sequence (*n* = 41,930), and probes and samples with unreliable measurements (detection *p* values >0.01) (*n* = 11,680 probes and 7 samples) were removed. Background subtraction was performed using noob.methylumi [55], and *β* values (ratio of methylated signal divided by the sum of the methylated and unmethylated signal) were normalized using BMIQ [56]. Finally, probes located on the sex chromosomes (*n* = 9489) and non-CpG probes (*n* = 2458) were removed, resulting in a final data set consisting of 390,787 probes and 377 samples. Cell-type proportions (CD8^+^ and CD4^+^ T-lymphocytes, natural killer cells, B cells, monocytes, and granulocytes) for each sample were estimated using the Houseman reference-based approach [57] and a suitable validated cord blood reference data set (R package *FlowSorted.CordBloodNorway.450K*), which we have recently established [47].

#### Differential DNA methylation analyses

All statistical tests were performed on the *M* values (log2 of the *β* values), which are more statistically valid for differential DNA methylation analyses than *β* values [58]. To identify differentially methylated positions associated with paracetamol and/or ADHD, we fitted a linear regression model using the limma package [59] and the mean DNA methylation differences.

We performed three sets of analyses to explore the relationship between prenatal exposure to paracetamol and DNA methylation differences in children with ADHD (Table 1). First, we stratified the analyses on paracetamol exposure and ADHD separately to evaluate the associations with DNA methylation. Second, to investigate whether prenatal exposure to paracetamol is associated with differences in DNA methylation in children with ADHD, we compared exposed samples with ADHD (synergistic effect group) to controls. Theoretically, significant associations from this comparison reflect both associations with ADHD and paracetamol. Hence, we also performed comparisons of this group to unexposed samples with ADHD (ADHD control group) as well as exposed samples without ADHD (drug exposure group) for interpretation purposes. Third, we divided the exposed samples with ADHD (synergistic effect group) into *sporadic* (6–19 days, *n* = 77 samples) or *long-term* (≥20 days, *n* = 19 samples) groups and used similar comparison groups as above. This enabled exploration of the epidemiological evidence that long-term exposure during pregnancy is associated with development of ADHD [18–22].

All analyses were conducted on the CpG site and region levels. Regional analysis was performed on predefined genomic regions (5 kb tiles, genes, promoters and CpG islands). Briefly, aggregated *p* values for each region were obtained by combining the uncorrected *p* values for each CpG in the region [53]. To adjust for multiple testing, a false discovery rate (FDR) cutoff of less than 5% was used for genome-wide significance by using the method of Benjamini and Hochberg (BH) [60]. Sex, smoking, maternal age, gestational age, estimated cell-type composition, BeadChip, and bisulfite conversion plate were included as covariates in the statistical model.

Analysis was also done based on a combined score of statistical significance and effect size to increase the biological relevance of the observed differences in DNA methylation as implemented in RnBeads [53]. Briefly, (1) the absolute difference in group means, (2) the quotient in group means, and (3) the *p* values from the linear model were ranked for all sites. Then, the rank for each of the three measures was aggregated into a combined score. The differentially methylated CpGs and regions were ranked according to this combined score.

#### Surrogate variable analyses

Surrogate variable analyses were performed to search for unmeasured sources of variation in DNA methylation confounding DNA methylation differences between groups. Specifically, surrogate variables were estimated directly from the DNA methylation data using the *sva* R package [61] with default parameters and group comparisons as targets.

#### Gene ontology analysis

Gene ontology (GO) analysis was performed using the *GOstats* package based on the top 100 differentially methylated genes ranked according to the combined score (effect size). Both over- and under-representation of GO terms were tested with a standard hypergeometric test among genes showing differential DNA methylation.

## Additional files

Additional file 1: Table S1. Significantly methylated CpGs between long-term exposed samples with ADHD and controls. (XLSX 1646 kb)

Additional file 2: Table S2. Significantly methylated CpGs between long-term exposed samples with ADHD and sporadically exposed samples with ADHD. (XLSX 585 kb)

Additional file 3: Table S3. Significantly methylated CpGs between long-term exposed samples with ADHD and unexposed samples with ADHD. (XLSX 101 kb)

Additional file 4: Figure S1. Enrichment of small *p* values associated with differences in DNA methylation in long-term exposed children with ADHD. Q-Q plots of the observed versus expected *p* values from the comparisons DNA methylation in cord blood from long-term exposed children with ADHD (synergy group, ≥20 days) to controls (blue, lambda = 2.2), sporadically exposed children with ADHD (red, lambda = 1.8) and unexposed children with ADHD (green, lambda = 1.7). The corresponding histograms of the nominal *p* values for each of the three comparisons display an enrichment of small *p* values. (PDF 887 kb)

## Abbreviations

ADHD: Attention-deficit/hyperactivity disorder
BH: Benjamini and Hochberg
DSM: Diagnostic and statistical manual
EWAS: Epigenome-wide association study
FDR: False discovery rate
GO: Gene ontology
GWAS: Genome-wide association study
HKD: Hyperkinetic disorder
ICD-10: 10th revision of the international classification of disease
MBRN: Medical birth registry of Norway
NPR: Norwegian patient registry
SVA: Surrogate variable analysis

## References

[1] Polanczyk GV, Salum GA, Sugaya LS, Caye A, Rohde LA (2015). Annual research review: a meta-analysis of the worldwide prevalence of mental disorders in children and adolescents. J Child Psychol Psychiatry.

[2] Faraone SV, Perlis RH, Doyle AE, Smoller JW, Goralnick JJ, Holmgren MA (2005). Molecular genetics of attention-deficit/hyperactivity disorder. Biol Psychiatry.

[3] Lesch K-P, Timmesfeld N, Renner TJ, Halperin R, Röser C, Nguyen TT (2008). Molecular genetics of adult ADHD: converging evidence from genome-wide association and extended pedigree linkage studies. J Neural Transm Springer Vienna.

[4] Neale BM, Su J, Anney R, Franke B, Zhou K, Maller JB (2008). Genome-wide association scan of attention deficit hyperactivity disorder. American journal of medical genetics. Part B, neuropsychiatric genetics: the official publication of the International Society of Psychiatric Genetics. NIH Public Access.

[5] Mick E, Todorov A, Smalley S, Hu X, Loo S, Todd RD (2010). Family-based genome-wide association scan of attention-deficit/hyperactivity disorder. Journal of the American Academy of Child & Adolescent Psychiatry.

[6] Neale BM, Medland S, Ripke S, Anney RJL, Asherson P, Buitelaar J (2010). Case-control genome-wide association study of attention-deficit/hyperactivity disorder. Journal of the American Academy of Child & Adolescent Psychiatry..

[7] Hinney A, Scherag A, Jarick I, Albayrak Ö, Pütter C, Pechlivanis S (2011). Genome-wide association study in German patients with attention deficit/hyperactivity disorder. American journal of medical genetics part B: neuropsychiatric genetics. Wiley Subscription Services, Inc A Wiley Company.

[8] Stergiakouli E, Hamshere M, Holmans P, Langley K, Zaharieva I, deCODE Genetics (2012). Investigating the contribution of common genetic variants to the risk and pathogenesis of ADHD. AJP.

[9] Zayats T, Athanasiu L, Sønderby IE, Djurovic S, Westlye LT, Tamnes CK, et al. Genome-wide analysis of attention deficit hyperactivity disorder in Norway. Yao Y-G, editor. PLoS One. Public Library of Science (PLoS); 2015; 10: e0122501. 10.1371/journal.pone.0122501 PMC4395400 25875332

[10] Neale BM, Medland SE, Ripke S, Asherson P, Franke B, Lesch K-P (2010). Meta-analysis of genome-wide association studies of attention-deficit/hyperactivity disorder. Journal of the American Academy of Child & Adolescent Psychiatry.

[11] Akutagava-Martins GC, Rohde LA, Hutz MH (2016). Genetics of attention-deficit/hyperactivity disorder: an update. Expert Review of Neurotherapeutics Taylor & Francis.

[12] Thapar A, Cooper M. Attention deficit hyperactivity disorder. Lancet; . Elsevier 2016; 387:1240–1250. 10.1016/S0140-6736(15)00238-X 26386541

[13] van Mil NH, Steegers-Theunissen RPM, Bouwland-Both MI, Verbiest MMPJ, Rijlaarsdam J, Hofman A (2014). DNA methylation profiles at birth and child ADHD symptoms. J Psychiatr Res.

[14] Xu Y, Chen X-T, Luo M, Tang Y, Zhang G, Wu D (2015). Multiple epigenetic factors predict the attention deficit/hyperactivity disorder among the Chinese Han children. J Psychiatr Res.

[15] Wilmot B, Fry R, Smeester L, Musser ED, Mill J, Nigg JT (2015). Methylomic analysis of salivary DNA in childhood ADHD identifies altered DNA methylation in VIPR2. J Child Psychol Psychiatry.

[16] Park S, Lee JM, Kim JW, Cho DY, Yun HJ, Han DH (2015). Associations between serotonin transporter gene (SLC6A4) methylation and clinical characteristics and cortical thickness in children with ADHD. Psychol Med.

[17] Walton E, Pingault JB, Cecil CAM, Gaunt TR, Relton CL, Mill J (2017). Epigenetic profiling of ADHD symptoms trajectories: a prospective, methylome-wide study. Mol Psychiatry.

[18] Liew Z, Ritz B, Rebordosa C, Lee P-C, Olsen J (2014). Acetaminophen use during pregnancy, behavioral problems, and hyperkinetic disorders. JAMA Pediatr.

[19] Thompson JMD, Waldie KE, Wall CR, Murphy R, Mitchell EA, the ABC study group (2014). Associations between acetaminophen use during pregnancy and ADHD symptoms measured at ages 7 and 11 years. Hashimoto K, editor. PLoS One.

[20] Avella-Garcia CB, Julvez J, Fortuny J, Rebordosa C, García-Esteban R, Galán IR, et al. Acetaminophen use in pregnancy and neurodevelopment: attention function and autism spectrum symptoms. Int J Epidemiol; Oxford University Press 2016; 6:1987-1996. 10.1093/ije/dyw115 27353198

[21] Stergiakouli E, Thapar A, Smith GD (2016). Association of acetaminophen use during pregnancy with behavioral problems in childhood: evidence against confounding. JAMA Pediatr.

[22] Brandlistuen RE, Ystrom E, Nulman I, Koren G, Nordeng H (2014). Prenatal paracetamol exposure and child neurodevelopment: a sibling-controlled cohort study. Int J Epidemiol.

[23] Lupattelli A, Spigset O, Twigg MJ, Zagorodnikova K, Mårdby AC, Moretti ME (2014). Medication use in pregnancy: a cross-sectional, multinational web-based study. BMJ Open.

[24] Elia J, Glessner JT, Wang K, Takahashi N, Shtir CJ, Hadley D (2011). Genome-wide copy number variation study associates metabotropic glutamate receptor gene networks with attention deficit hyperactivity disorder. - PubMed-NCBI. Nat Genet.

[25] Kitagishi Y, Minami A, Nakanishi A, Ogura Y, Matsuda S (2015). Neuron membrane trafficking and protein kinases involved in autism and ADHD. International journal of molecular sciences. Multidisciplinary Digital Publishing Institute (MDPI).

[26] Ballas N, Grunseich C, Lu DD, Speh JC, Mandel G. REST and its corepressors mediate plasticity of neuronal gene chromatin throughout neurogenesis. Cell; Elsevier 2005; 121:645–657. 10.1016/j.cell.2005.03.013 15907476

[27] Wakatsuki S, Furuno A, Ohshima M, Araki T. Oxidative stress-dependent phosphorylation activates ZNRF1 to induce neuronal/axonal degeneration. J Cell Biol; Rockefeller Univ Press 2015; 211:881–896. 10.1083/jcb.201506102 PMC4657170 26572622

[28] Tam S-Y, Lilla JN, Chen C-C, Kalesnikoff J, Tsai M. RabGEF1/Rabex-5 regulates TrkA-mediated neurite outgrowth and NMDA-induced Signaling activation in NGF-differentiated PC12 cells. Obukhov AG, editor. PLoS One; Public Library of Science 2015; 10: e0142935. 10.1371/journal.pone.0142935 PMC4654474 26588713

[29] Cubelos B, Sebastián-Serrano A, Beccari L, Calcagnotto ME, Cisneros E, Kim S, et al. Cux1 and Cux2 regulate dendritic branching, spine morphology, and synapses of the upper layer neurons of the cortex. Neuron; Elsevier 2010; 66:523–535. 10.1016/j.neuron.2010.04.038 PMC2894581 20510857

[30] Medrihan L, Cesca F, Raimondi A, Lignani G, Baldelli P, Benfenati F (2013). Synapsin II desynchronizes neurotransmitter release at inhibitory synapses by interacting with presynaptic calcium channels. Nat Comms.

[31] Calabrese V, Mancuso C, Calvani M, Rizzarelli E, Butterfield DA, Stella AMG (2007). Nitric oxide in the central nervous system: neuroprotection versus neurotoxicity. Nat Rev Neurosci.

[32] Imbrici P, Camerino DC, Tricarico D (2013). Major channels involved in neuropsychiatric disorders and therapeutic perspectives. Front Genet Frontiers Media SA.

[33] Philippot G, Nyberg F, Gordh T, Fredriksson A, Viberg H (2016). Short-term exposure and long-term consequences of neonatal exposure to Δ9-tetrahydrocannabinol (THC) and ibuprofen in mice. Behav Brain Res.

[34] Viberg H, Eriksson P, Gordh T, Fredriksson A. Paracetamol (acetaminophen) administration during neonatal brain development affects cognitive function and alters its analgesic and anxiolytic response in adult male mice. Toxicol Sci; Oxford University Press 2014; 138:139–147. 10.1093/toxsci/kft329 24361869

[35] Mooney MA, McWeeney SK, Faraone SV, Hinney A, Hebebrand J, IMAGE2 Consortium (2016). Pathway analysis in attention deficit hyperactivity disorder: an ensemble approach. Am J Med Genet B Neuropsychiatr Genet.

[36] Guney E, Cetin FH, Alisik M, Tunca H, Tas Torun Y, Iseri E (2015). Attention deficit hyperactivity disorder and oxidative stress: a short term follow up study. Psychiatry Res.

[37] Archana E, Pai P, Prabhu BK, Shenoy RP, Prabhu K, Rao A (2011). Altered biochemical parameters in saliva of pediatric attention deficit hyperactivity disorder. Neurochem Res Springer US.

[38] Ceylan MF, Sener S, Bayraktar AC, Kavutcu M (2012). Changes in oxidative stress and cellular immunity serum markers in attention-deficit/hyperactivity disorder. Psychiatry Clin Neurosci.

[39] Goldani AAS, Downs SR, Widjaja F, Lawton B, Hendren RL. Biomarkers in Autism. Frontiers in Psychiatry. Frontiers Media SA; 2014; 5:950. 10.3389/fpsyt.2014.00100 PMC4129499 25161627

[40] Popa-Wagner A, Mitran S, Sivanesan S, Chang E, Buga A-M (2013). ROS and brain diseases: the good, the bad, and the ugly. Oxidative Med Cell Longev.

[41] Masutani H (2001). Oxidative stress and redox imbalance in acetaminophen toxicity. The Pharmacogenomics Journal.

[42] Ghanizadeh A, Bahrani M, Miri R, Sahraian A (2012). Smell identification function in children with attention deficit hyperactivity disorder. Psychiatry Investigation Korean Neuropsychiatric Association.

[43] Romanos M, Renner TJ, Schecklmann M, Hummel B, Roos M, Mering von C (2008). Improved odor sensitivity in attention-deficit/hyperactivity disorder. Biol Psychiatry.

[44] Weiland R, Macht M, Ellgring H, Groß-Lesch S, Lesch K-P, Pauli P (2011). Olfactory and gustatory sensitivity in adults with attention-deficit/hyperactivity disorder. Atten Defic Hyperact Disord.

[45] Ystrom E, Vollrath ME, Nordeng H. Effects of personality on use of medications, alcohol, and cigarettes during pregnancy. Eur J Clin Pharmacol; Springer-Verlag 2011; 68:845–851. 10.1007/s00228-011-1197-y 22189674

[46] Kalda A, Zharkovsky A. Epigenetic mechanisms of Psychostimulant-induced addiction. Int Rev Neurobiol. 2015:85–105. 10.1016/bs.irn.2015.02.010 26070754

[47] Gervin K, Page CM, Aass HCD, Jansen MA, Fjeldstad HE, Andreassen BK (2016). Cell type specific DNA methylation in cord blood: a 450K-reference data set and cell count-based validation of estimated cell type composition. Epigenetics..

[48] Magnus P, Birke C, Vejrup K, Haugan A, Alsaker E, Daltveit AK, et al. Cohort profile update: the Norwegian mother and child cohort study (MoBa). Int J Epidemiol; . Oxford University Press 2016:1-7. 10.1093/ije/dyw029 27063603

[49] Rønningen KS, Paltiel L, Meltzer HM, Nordhagen R, Lie KK, Hovengen R (2006). The biobank of the Norwegian mother and child cohort study: a resource for the next 100 years. Eur J Epidemiol.

[50] Paltiel L, Haugan A, Skjerden T, Harbak K, Bækken S, Stensrud NK, et al. The biobank of the Norwegian mother and child cohort study—present status. Norsk Epidemiologi. 2014;24:29–35.

[51] Taylor E, Schachar R, Thorley G, Wieselberg HM, Everitt B, Rutter M. Which boys respond to stimulant medication? A controlled trial of methylphenidate in boys with disruptive behaviour. Psychol Med; Cambridge University Press 1987; 17:121–143. 10.1017/s0033291700013039 3554290

[52] Thapar A, Pine DS, Leckman JF, Scott S, Snowling MJ, Taylor E. Rutter’s child and adolescent psychiatry. Chichester, UK: Wiley; 2015.

[53] Assenov Y, Müller F, Lutsik P, Walter J, Lengauer T, Bock C (2014). Comprehensive analysis of DNA methylation data with RnBeads. Nat Meth Nature Publishing Group.

[54] Chen Y-A, Lemire M, Choufani S, Butcher DT, Grafodatskaya D, Zanke BW (2014). Discovery of cross-reactive probes and polymorphic CpGs in the Illumina Infinium HumanMethylation450 microarray. Epigenetics.

[55] Triche TJ, Weisenberger DJ, Van Den Berg D, Laird PW, Siegmund KD (2013). Low-level processing of Illumina Infinium DNA Methylation BeadArrays. Nucleic Acids Res.

[56] Teschendorff AE, Marabita F, Lechner M, Bartlett T, Tegner J, Gomez-Cabrero D (2013). A beta-mixture quantile normalization method for correcting probe design bias in Illumina Infinium 450 k DNA methylation data. Bioinformatics.

[57] Houseman E, Accomando WP, Koestler DC, Christensen BC, Marsit CJ, Nelson HH (2012). DNA methylation arrays as surrogate measures of cell mixture distribution. BMC Bioinformatics.

[58] Du P, Zhang X, Huang C-C, Jafari N, Kibbe WA, Hou L (2010). Comparison of Beta-value and M-value methods for quantifying methylation levels by microarray analysis. BMC Bioinformatics BioMed Central.

[59] Ritchie ME, Phipson B, Wu D, Hu Y, Law CW, Shi W (2015). Limma powers differential expression analyses for RNA-sequencing and microarray studies. Nucleic Acids Res.

[60] Benjamini Y. Discovering the false discovery rate. Journal of the Royal Statistical Society: series B (statistical methodology); Blackwell Publishing Ltd; 2010; 72:405–416.

[61] Leek JT, Johnson WE, Parker HS, Jaffe AE, Storey JD. The sva package for removing batch effects and other unwanted variation in high-throughput experiments. Bioinformatics; . Oxford University Press 2012; 28:882–883. 10.1093/bioinformatics/bts034 PMC3307112 22257669

