# Supplementary figures and images for: Long-term prenatal exposure to paracetamol is associated with DNA methylation differences in children diagnosed with ADHD
Source: Clin Epigenetics. 2017 Aug 2;9:77. doi: 10.1186/s13148-017-0376-9 (PMC5540511; doi:10.1186/s13148-017-0376-9)

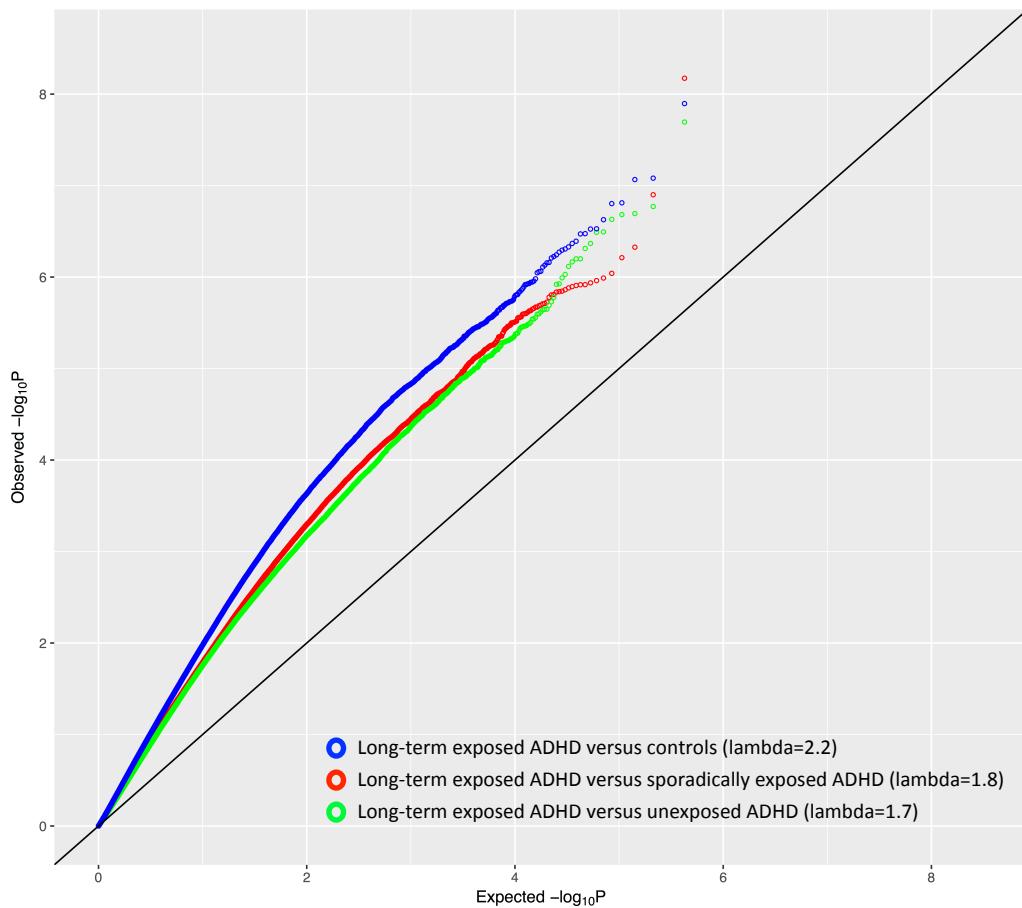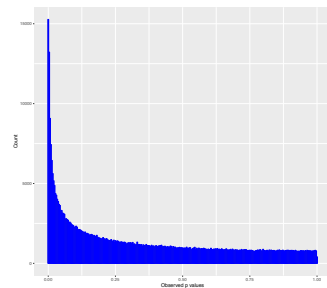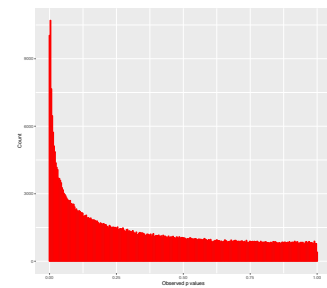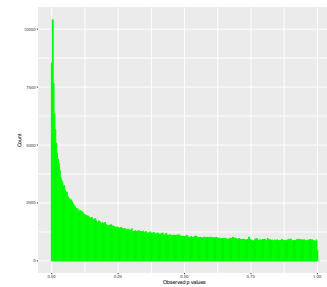

Supplement: Supplementary file 4 — Enrichment of small p values associated with differences in DNA methylation in long-term exposed children with ADHD. Q-Q plots of the observed versus expected p values from the comparisons DNA methylation in cord blood from long-term exposed children with ADHD (synergy group, ≥20 days) to controls (blue, lambda = 2.2), sporadically exposed children with ADHD (red, lambda = 1.8) and unexposed children with ADHD (green, lambda = 1.7). The corresponding histograms of the nominal p values for each of the three comparisons display an enrichment of small p values. (PDF 887 kb) [file 13148_2017_376_MOESM4_ESM.pdf]
